# Clinical Presentation, Diagnosis, and Management of Wrist Intravascular Papillary Endothelial Hyperplasia: A Case Report and Literature Review

**DOI:** 10.7759/cureus.70539

**Published:** 2024-09-30

**Authors:** Abdulrahim S Alrasheed, Ibrahim M Aldandan, Sadaf Noor, Abdulrahman Al-Schameri

**Affiliations:** 1 Department of Neurosurgery, College of Medicine, King Faisal University, AlAhsa, SAU; 2 Department of Laboratory Medicine, Almoosa Specialist Hospital, AlAhsa, SAU; 3 Department of Neurosurgery, Almoosa Specialist Hospital, AlAhsa, SAU

**Keywords:** hand, intravascular papillary endothelial hyperplasia (masson's tumor), neuropathy, soft tissue lesion, vascular malformation, wrist

## Abstract

Intravascular papillary endothelial hyperplasia (IPEH), or Masson's tumor, is a benign vascular mass that is often misdiagnosed due to its nonspecific clinical signs. The majority of IPEH cases involve blood vessel thrombosis. Although it is a rare tumor, IPEH requires an accurate diagnosis to avoid unnecessary treatment. Overall, the tumor generally has a good prognosis. In this case report, a 16-year-old male with a known four-year history of right wrist swelling and digit numbness presented to the outpatient clinic due to worsening pain and swelling for the previous month. Based on the physical examination and radiological examination, our preliminary diagnosis was either schwannoma or neurofibroma, for which a total surgical excision was done. Postoperatively, the tumor was examined histologically and revealed a benign vascular lesion with no atypical features, which were consistent with IPEH. To prevent unnecessary invasive operations and radiation, surgeons must be aware of this rare tumor. The following case report emphasizes the importance of differentiation between Masson`s hemangioma and other similar tumors to prevent unnecessary invasive operations and radiation.

## Introduction

Intravascular papillary endothelial hyperplasia (IPEH), or what is known as Masson’s tumor, is a slowly growing rare benign vascular mass of the skin and subcutis tissues, accounting for less than 2% of soft tissue masses [1]. Its clinical presentation is not specific [2]. Pierre Masson initially described it as an intravascular growth that develops within the lumen of an inflamed hemorrhoidal plexus [1]. IPEH tumors are marked by the presence of multiple small papillary structures that are covered with endothelial cells and feature hyaline cores [3]. Although it originates either from vascular malformation or normal blood vessels, there are uncertainties about the pathogenesis of the disease. However, it is commonly associated with the formation of a thrombus in the vessel lumens [3,4]. The challenge is that it usually comes out as a misdiagnosed case, such as angiosarcoma, because it resembles other conditions clinically, especially when it occurs in the hand [4]. In such cases, the prognosis is very good. The differentiation from other causes, however, is necessary to avoid unneeded invasive procedures and radiation [1]. Through this report and our review of the literature, we aim to facilitate the identification of this tumor from other conditions.

## Case presentation

**Figure 1:**
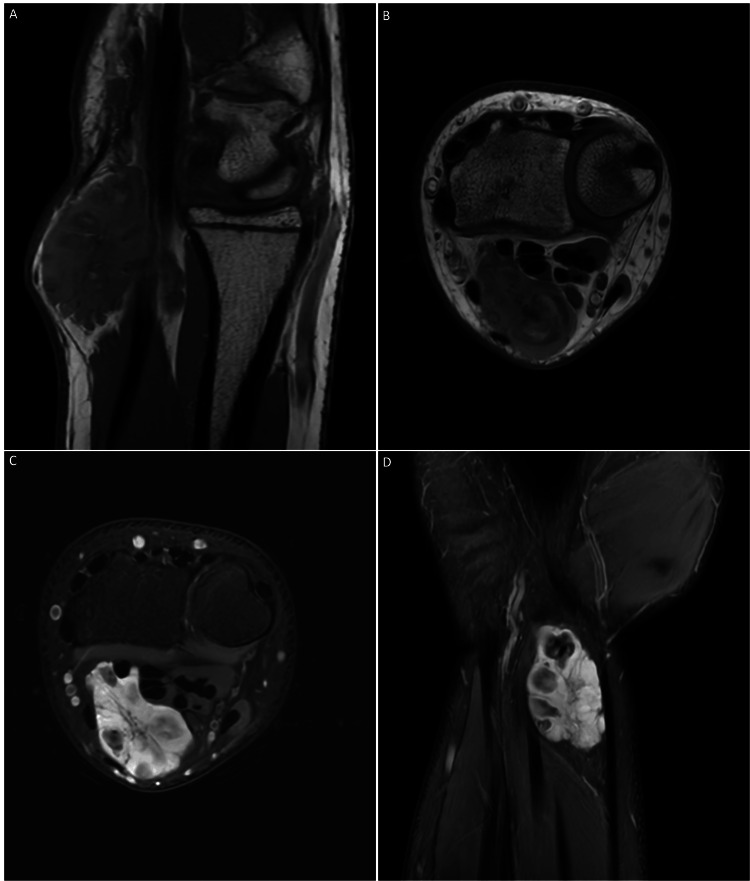
Preoperative imaging findings A and B: Right wrist lobulated ventral mass is seen superficial to the flexor tendon sheath and eccentric to the median nerve. It displaces the median nerve to the ulnar side and insinuates between the flexor digitorum and flexor pollicis longus. The mass measures around 4×2×1.5 cm. It shows well-defined borders without invasion of the muscles or tendons. C: On T2 weighted imaging, it demonstrates central low signal intensity and peripheral bright signal intensity in keeping with the target sign, and it shows multiple rings in keeping with the fascicular sign. D: Fat septation within the mass is also seen. Heterogenous enhancement is noted through the mass. There is no intramuscular component. The underlying bones are intact.

A 16-year-old male with unremarkable surgical, medical, psychosocial, or family history had swelling in the right anterior aspect of the wrist for four years. He presented to the outpatient clinic due to worsening pain and swelling that had been interfering with his daily life activities for the past month. On physical examination, there was a 4 cm, firm, dark yellowish, painful lump on the right anterior aspect of the forearm that was tender on palpation. There were no signs of bruise-like patches in the overlying or adjacent skin. There was a firm lesion surface, with an unremarkable change in the adjacent skin. Numbness in the second to fifth digits was associated with the painful lump, and the Tinel sign was negative. No other similar lesions were identified on the left or right hand. Based on the lesion's clinical appearance, it was most likely to be schwannoma or neurofibroma. Ultrasonography revealed a 3.5 x 0.9 x 2 cm ill-defined soft tissue origin lesion. MRI showed lobulated eccentric ventral mass displacing the median nerve to the ulnar side (Figures 1A, 1B). There was a demonstration of the target sign and fascicular sign (Figure 1C). Heterogenous enhancement and fat septation were noted through the mass (Figure 1D). Therefore, it was most likely to be contained within the nerve sheath. Based on these findings, the preliminary diagnosis was either schwannoma or neurofibroma. 

**Figure 2:**
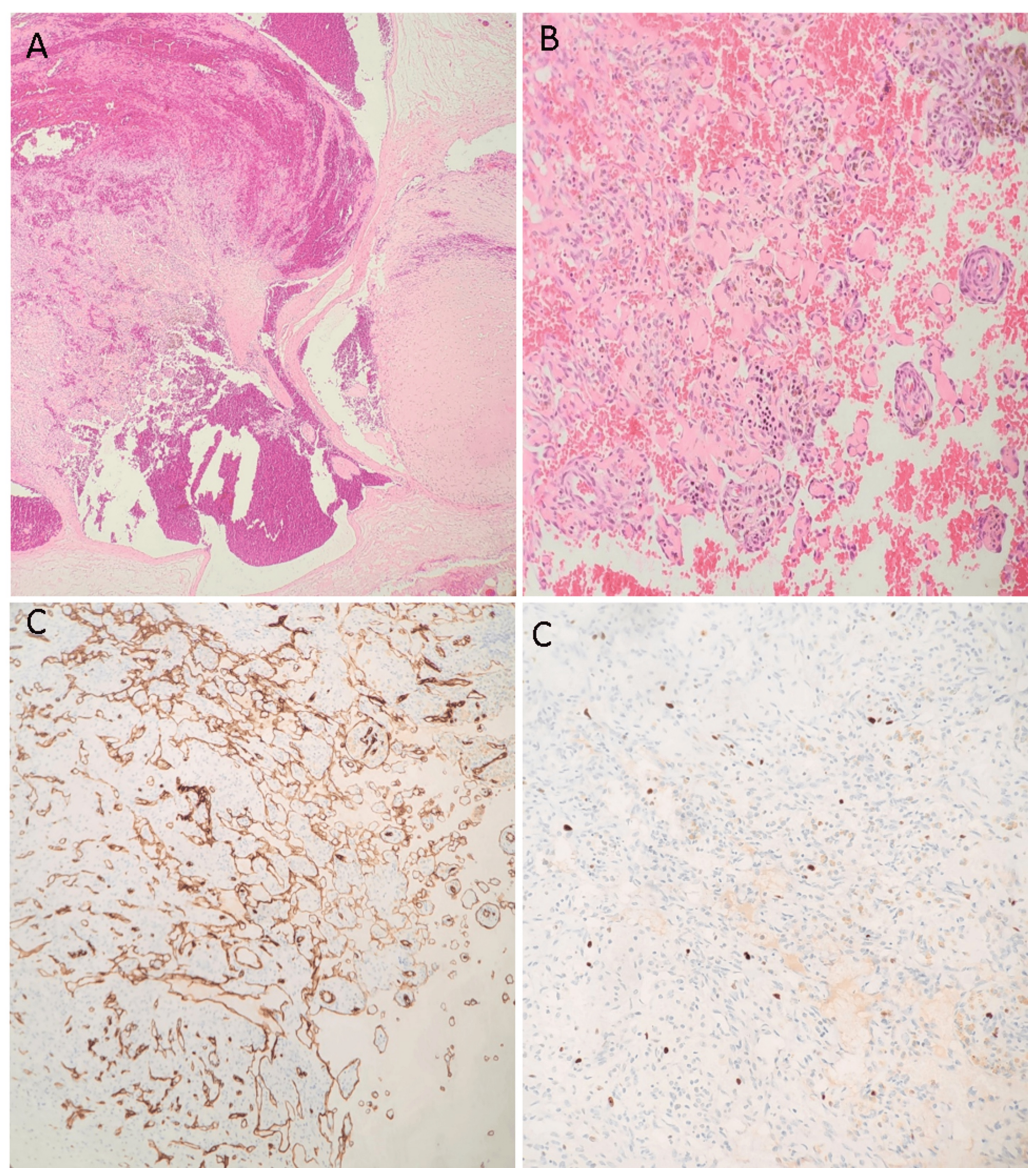
Histopathological findings A: A blood vessel with papillary proliferation of endothelial cells and thrombus formation at the center (hematoxylin and eosin, ×10). B: Papillary proliferation of endothelial cells forming papillary structures with hyalinized cores lined by a single layer of cytologically bland cells (hematoxylin and eosin, ×40). C: CD34 immunohistochemical stain highlighted the vascular endothelial cells. D: Ki67 / MIB 1 immunohistochemical stain demonstrating a very low proliferating index.

Our patient was brought to the operating theater, underwent general anesthesia, and, via a vertical incision, a complete excision was done, and the lesion was sent for histopathology. During the operation, the mass was found to be highly vascularized. No complications were faced during or after wound closure. The gross examination of the mass revealed gray-brown nodular soft tissue with a lobulated external surface measuring 5 x 3 x 2 cm. Sectioning revealed multiple cyst-like spaces filled with blood and thrombus. Microscopic examination revealed a circumscribed vascular lesion comprised of dilated vessels with an associated intravascular papillary proliferation of plump endothelial cells without atypia and with fibrin deposition. A high-power view showed small hyalinized cores lined by endothelial cells (Figure 2A). No infiltrative growth pattern, mitosis, or necrosis was seen (Figure 2B). To support the vascular nature, the endothelial cells were highlighted by CD34 immunohistochemical staining (Figure 2C). A very low proliferative index was found by using the Ki67 immunohistochemistry staining, with the positive nuclei mostly representing rare scattered inflammatory cells (Figure 2D). This excluded high-grade vascular tumors, which can mimic Masson’s hemangioma morphologically. 

After five months of serial clinical follow-up, the patient recovered well postoperatively and showed no symptoms of tumor recurrence. To the best of our knowledge, this is the first case of wrist Masson's hemangioma in Saudi Arabia. The need for ethical approval was waived due to the study design. A written consent regarding the publication of this case was signed by the patient’s guardian.

## Discussion

IPEH accounts for less than 2% of soft tissue masses [5]. Typically, the lesions range in diameter from 0.2 to 2 cm, are sharply demarcated, and predominantly occur in females (57% female versus 43% male) [6,7]. The common age of its presentation is within the third and fourth decades of life [8]. Its usual presentation is a small, firm, superficial mass that slowly grows. Besides, the overlying skin could become blue or red. The most common locations are hands, fingers, head, and neck, respectively [1,9]. It is usually of three types: 1. a pure form presents with a small subcutaneous mass, frequently in fingers in a dilated vessel; 2. a mixed form that occurs as a change in a vascular malformation (the most common); 3. an undetermined form that has an extravascular origin and evolves from a pre-existing hematoma [3,5,9]. In our case, the patient was in his second decade of life, and the lesion’s overlying skin was dark yellowish in color. Furthermore, the lesion’s type was the first type, which is a pure form.

Multiple theories have been discussed regarding the pathogenesis of the lesion. A hormonal etiology was suggested by a previous study due to its resemblance to other benign vascular masses. Another theory defines the role of fibroblast growth factor beta due to its ability to induce thrombus formation and endothelial proliferation. A close association with thrombus or thrombotic material has been suggested to be present in all cases [6]. Other studies suggest that trauma has a major role [5]. The female predominance and the excessive proliferation during pregnancy reports suggest a hormonal association as well [6]. Diagnosing IPEH patients is challenging because of the presence of nonspecific clinical imaging findings; hence, the gold standard for diagnosing IPEH is histological examination [5,10]. The appearance of intravascular papillary structures covered by hyperplastic endothelial cells is a significant histopathological feature of IPEH. Despite the extensive literature review, only four cases have been reported to be in the wrist (Table 1). Since the lesion has a close association with thrombus or thrombotic material in most cases, the presence of an organizing thrombus gives a clue to the diagnosis [6,8]. Our case showed features consistent with these.

**Table 1:** Demographic, clinical, histopathological, and result of treatment for previously published cases of IPEH F: female; Y: years; N/A: not applicable

Author	Age, Sex	Clinical signs	Site	Histological findings	Surgical resection	Outcome
Shah et al., 2023 [5]	37y, F	Nerve-like pain Numbness Tingling	Wrist, median nerve peripheral sheath	Multiple irregularly contoured vascular caverns lined by endothelial cells with dense fibrous connective tissue septae	Complete	Asymptomatic at two months of follow up
Titchener et al., 2015 [11]	43y, F	Progressive paraesthesia Progressive pain	Wrist, between the deep and superficial flexor tendons distal to the carpal tunnel	Multiple fragments of fibro-collagenous tissue containing vascular channels Multiple small papillary structures with hyalinised fibrinoid material in the papillary cores Inflammation and fibrosis surrounding the vascular channels	Complete	A postoperative satisfactory recovery
Schwartz et al., 2008 [12]	70y, F	Palpable progressive mass	Wrist, right dorsal aspect	Multiple irregular endothelial-lined vascular channels and papillary structures	Complete	N/A
Leilei et al., 2024 [13]	47y, M	Mild pain	Wrist, right dorsal aspect	Papillary hyperplasia of vascular endothelial cells, with a core of fibrous connective tissue	Compelte	No recurrence

Detection of associated vessels by the use of ultrasound may help in differentiating IPEH from other soft tissue masses [10]. Although the histological examination is the gold standard for diagnosing IPEH, the accurate interpretation of the histological findings is of high importance due to its similarity to other neurological and vascular tumors like neurofibroma, angiosarcoma, Kaposi sarcoma, neurogenic tumors, and glomus tumors [8]. It is highly essential to differentiate between IPEH and other tumors, especially malignancies, to abstain from unnecessary surgery and radiation. Simple excision is usually curative [1]. Although almost all cases of Masson’s hemangioma present with vascular symptoms, in our case the patient presented with only neurogenic symptoms. Our patient was treated with a total surgical excision with no recurrences after five months of the surgery.

## Conclusions

Masson's hemangioma is a rare tumor with a favorable prognosis. However, it is a difficult tumor to diagnose due to its clinical resemblance to other tumors. In our case, a 16-year-old male presented with neurological symptoms that were attributed to neurofibroma. Thus, surgical excision and histological examination were obtained, which eventually confirmed Masson's hemangioma. The gold standard treatment for IPEH is total surgical excision, which poses a low risk of recurrence. Overall, IPEH should be kept in mind if a painful yellowish or bluish skin lesion is encountered. Reporting such cases is of great importance to enhance patient care and abstain from unnecessary invasive procedures.
